# Feed-forward loop improves the transient dynamics of an antithetic biological controller

**DOI:** 10.1098/rsif.2024.0467

**Published:** 2025-01-22

**Authors:** Thales R. Spartalis, Mathias Foo, Xun Tang

**Affiliations:** ^1^ Cain Department of Chemical Engineering, Louisiana State University, Baton Rouge, LA 70803, USA; ^2^ School of Engineering, University of Warwick, Coventry CV4 7AL, UK

**Keywords:** antithetic controller, feed-forward loop, computational analysis, gene circuits

## Abstract

Integral controller is widely used in industry for its capability of endowing perfect adaptation to disturbances. To harness such capability for precise gene expression regulation, synthetic biologists have endeavoured in building biomolecular (quasi-)integral controllers, such as the antithetic integral controller. Despite demonstrated successes, challenges remain with designing the controller for improved transient dynamics and adaptation. Here, we explore and investigate the design principles of alternative RNA-based biological controllers, by modifying an antithetic integral controller with prevalently found natural feed-forward loops (FFL), to improve its transient dynamics and adaptation performance. With model-based analysis, we demonstrate that while the base antithetic controller shows excellent responsiveness and adaptation to system disturbances, incorporating the type-1 incoherent FFL into the base antithetic controller could attenuate the transient dynamics caused by changes in the stimuli, especially in mitigating the undesired overshoot in the output gene expression. Further analysis on the kinetic parameters reveals similar findings to previous studies that the degradation and transcription rates of the circuit RNA species would dominate in shaping the performance of the controllers.

## 1. Introduction

Building synthetic feedback control gene circuits for precise and robust regulation of a gene expression is a longstanding interest to synthetic biologists [1–7], as it is critical to achieving precise chemical and biological signals mediation, and protein production in coping with internal and external disturbances and unwarranted changes [8–10]. Natural systems provide numerous examples of robust perfect adaptation, including *Escherichia coli* chemotaxis and calcium homeostasis in mammalian cells [11]. In a feedback control scheme, the controller monitors the dynamics of the species of interest to determine the corresponding regulation of the process, and to mitigate or eliminate the impact of disturbances and uncertainties, for improved control precision and robustness. Since synthetic systems often exhibit vulnerabilities to fluctuations in the surrounding environment, the design and implementation of a robust controller are thus essential. This has led to a variety of work on designing and implementing synthetic feedback controllers for cell population regulation, biofuel production and single-cell gene expression regulation [12–16].

Previous studies demonstrated that the feedback mechanism in a biological system can be achieved with molecular sequestration, using proteins, such as transcription factors, or nucleic acids, such as RNA and antisense RNA [17–20]. Among different feedback controllers, integral controllers are of special interest, as they promise perfect adaptation theoretically. Briat *et al.* introduced an antithetic integral controller [5], which employs a sequestration reaction between two controller species, where one actuates the system dynamics and the other is influenced by the system output, to achieve an integral controller design for reference tracking and disturbance adaptation [2]. This mechanism enables the circuit to adapt by showcasing its potential for reference tracking and perfect adaptation to environmental disturbances [2,5,6,8,9,21]. Similar studies include Shopera *et al*., which presented a negative feedback-based transcription controller to adapt for ribosome fluctuation [22]; Agrawal *et al*. leveraged $\sigma_{28}$ and anti-$\sigma_{28}$ for molecular sequestration to realize an integral controller [23]; Cuba Samaniego & Franco [3] deployed an activation–deactivation reaction cycle and a molecular sequestration to realize an ultrasensitive quasi-integral controller; as well as related works from the Del Vecchio’s group [24–27]. However, despite the prosperity of studies on feedback controllers, there still exist limitations to overcome for enhanced reliability and practical implementations. For example, as demonstrated in [6,9,21], the transient dynamics of the antithetic controller need to be further improved for a rapid response to stimuli, and the perfect adaptation requires delicate tuning of the kinetic parameters [8]. Besides, understanding the dynamics of the biological system is also critical to designing effective biological controllers. Therefore, vast research efforts exist where multi-omics kinetic models are used to investigate the dynamic behaviour of the biological systems [28–30]. In [31], the authors integrated multiple omics data sources and developed a multi-omics model to predict the genome-wide concentrations and growth dynamics in *E. coli* cells. By incorporating multiple omics layers and known gene regulatory and protein–protein interactions, the model improves the prediction accuracy, providing a broadly applicable framework for guiding biological discovery [31]. In a recent study [32], Di Filippo *et al*. developed a computational pipeline which leverages a constraint-based model to integrate metabolomics and transcriptomics data and to characterize multi-level metabolic regulation, considering both enzyme expression levels and metabolites interactions [32]. Finally, a modification to antithetic controllers with additional regulations is proposed in [33], where a biomolecular proportional-integral-derivative controller was proposed to improve the stability of genetic circuits and mitigate stochastic noise in yeast cells. Nevertheless, exploring alternative designs and further understanding the design principles of biological controllers remain an ongoing task.

**Figure 1.**
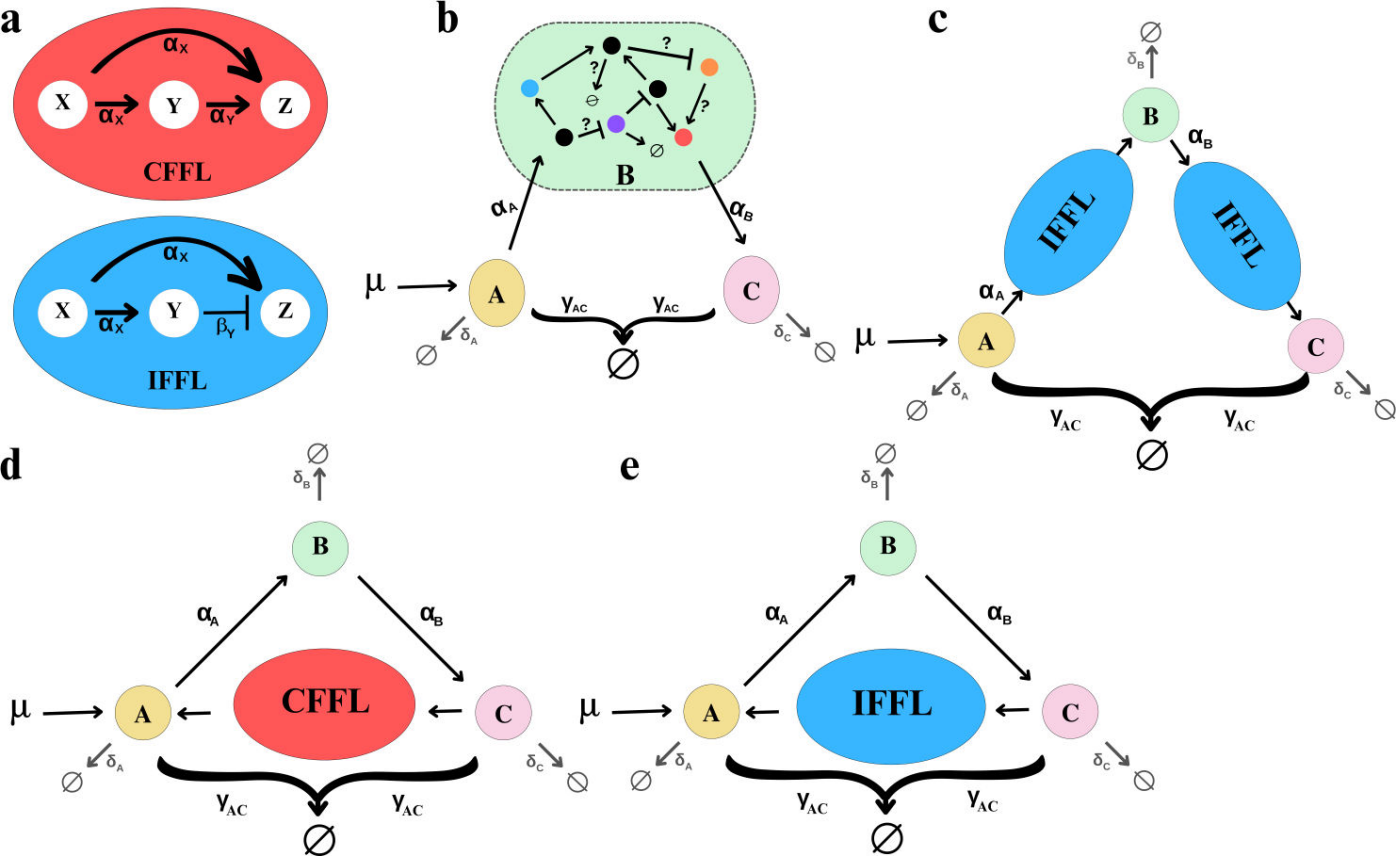
Schematics of different antithetic controller designs. (*a*) CFFL and IFFL are composed of three nodes *X*, *Y* and *Z*. (*b*) Base circuit with B representing a single gene expression process. (*c*) 2IFFL-based circuit. (*d*) CFFL-based circuit. (*e*) 1IFFL-based circuit.

Designing new biological controllers can be achieved with computational approaches, where given an objective function of the circuit dynamics, one can perform optimization to design the number of nodes (gene elements, such as plasmid, mRNA and protein) and the interactions, by minimizing the difference between the circuit output and the objective dynamics [34,35]. Alternatively, it can also be achieved with reasoning based on domain expertise, known dynamics and functions of existing regulatory parts and network motifs [18,36–38]. One of the most well-studied network motifs is the feed-forward loop (FFL), which is composed of three biological nodes, *X*, *Y* and *Z*, as shown in figure 1*a*. In the type-1 coherent feed-forward loop (CFFL), both *X* and *Y* would activate the expression of node *Z*, whereas in the type-1 incoherent feed-forward loop (IFFL), *X* activates *Z* expression, but *Y* represses *Z* expression. The CFFLs and IFFLs are prevalent in the transcriptional networks of bacterial cells [39–41]. For example, Mangan & Alon [42] presented the design of CFFL and IFFL circuits with natural motifs, using gene *fnr* and gene *arcA* as the *X* and *Y* species, respectively, while realizing a type-1 CFFL with gene *focA* as species *Z*, or a type-1 IFFL with gene *glpACB* as species *Z*. Given such prevalence in the biological systems, as well as their well-characterized properties and proven functions as signal amplifiers [42,43], noise filters [44], fold-change detector [45], perceptron [46] and pulse generation [47–49], here, in this study, we investigate the modification of a classical antithetic integral controller proposed by Briat *et al*. [5], with the FFLs, in improving the transient dynamics of the controller and attenuating the impact from disturbances.

Specifically, we conduct a simulation-based analysis of alternative designs of the antithetic controllers. We first analyse their characteristics with widely used parameter values in literature [5,6] without considering their biological feasibility, expecting to grasp a general understanding of each design. To complement the efforts, we then analyse the circuits with kinetic parameters previously fitted to experiments from previous works [50–53] to infer their practical applicability and limitations. The results indicate that proper incorporation of FFL motifs into the base antithetic controller could improve the adaptation and attenuate undesirable overshoot subject to disturbances.

## 2. Results and discussion

### 2.1. Circuits design and mathematical modelling

We first set off to define a base antithetic controller (hereinafter termed a base circuit) as depicted in figure 1*b*), as a benchmark to gauge the performance of our proposed circuits. This base circuit is composed of nodes *A*, *B* and *C*, where *A* and *C* are independent gene species, and *B* represents the biological system to be regulated. While *B* could contain a complex network as shown in the figure, for simplicity, here we consider a single gene expression process in *B*, for all the circuits introduced in this work. Note that, as we restrict our focus to RNA-based gene circuits, all the gene species described in the work refer to (m)RNA, unless otherwise specified. The production of gene *A* is activated by inducer μ, which then promotes gene *B* production at a transcription rate $\alpha_{A}$. Gene *B* promotes the transcription of gene *C* at a transcription rate $\alpha_{B}$. Genes *A* and *C* sequester each other at a sequestration rate of $\gamma_{AC}$, to form a feedback function. All genes degrade at their corresponding degradation rate δ. The detailed mechanistic ordinary differential equation (ODE) model is given in equation (2.1).


(2.1) $$\begin{array}{l} \frac{d\left[A\right]}{dt}=\;\mu -\;\gamma_{AC}\;.\;\left[A\right]\;.\;\left[C\right]\;-\;\delta_{A}\;.\;\left[A\right]\\ \frac{d\left[B\right]}{dt}=\;\alpha_{A}\;.\;\left[A\right]-\;\delta_{B}\;.\;\left[B\right]\\ \frac{d\left[C\right]}{dt}=\;\alpha_{B}\;.\;\left[B\right]\;-\;\gamma_{AC}\;.\;\left[A\right]\;.\;\left[C\right]-\;\delta_{C}\;.\;\left[C\right] \end{array}$$

We then explore antithetic controllers modified with type-1 CFFL and type-1 IFFL motifs. The type-1 CFFL is widely considered a sign-sensitive delay element, which means it shows a delay only for positive steps, thus acknowledged as an asymmetric filter [18]. The type-1 IFFL circuit is well recognized as a pulse generator, offering speed increase to dynamic responses of the system [49]. Therefore, we expect the incorporation of the type-1 CFFL and IFFL motifs would benefit the transient dynamics of the base circuit, such as accelerated response to the stimuli, rapid settling after the disturbance and moderate overshoot if there is any. Note that, while the number of the FFL motifs and the locations for them to be integrated into the base circuit would contribute to numerous architectures, we have confined our design to two motifs in the circuit, to avoid overly complex designs. Based on our preliminary investigation (electronic supplementary material, figures S1–S3), three designs showed the best performances in terms of both transient and steady-state dynamics.

Specifically, the first FFL-modified circuit is the CFFL-based antithetic controller, as shown in equation (2.2), and is named CFFL-based circuit throughout the rest of this manuscript. In comparison with the base model, one CFFL motif is introduced to establish a promotion pathway from gene *C* to gene *A*, where gene *C* directly promotes the transcription of gene *X* in the CFFL motif, and the CFFL motif output gene *Z* promotes the transcription of gene *A*. Such a design amplifies the sensitivity of gene *A* to changes in gene *C*, thus holding the promise of accelerating and strengthening the feedback response from the sequestration reaction. Within the CFFL, we assumed the same activation rate α from gene *X* to genes *Y* and *Z*, for simplicity, but we expect a customized activation/transcription rate for each species to deliver further improved dynamics for specific applications. On the other hand, all genes degrade at their corresponding degradation rate δ. The detailed ODE model of this circuit is shown in equation (2.2).


(2.2) $$\begin{array}{l} \frac{d\left[A\right]}{dt}=\;\mu +\alpha_{Z}.\;\left[Z\right]-\;\gamma_{AC}\;.\;\left[A\right]\;.\;\left[C\right]\;-\;\delta_{A}\;.\;\left[A\right]\\ \frac{d\left[B\right]}{dt}=\;\alpha_{A}\;.\;\left[A\right]-\;\delta_{B}\;.\;\left[B\right]\;\\ \frac{d\left[X\right]}{dt}=\;\alpha_{C}\;.\;\left[C\right]-\;\;\delta_{X}\;.\;\left[X\right]\;\\ \frac{d\left[Y\right]}{dt}=\;\alpha_{X}\;.\;\left[X\right]-\;\;\delta_{Y}\;.\;\left[Y\right]\\ \frac{d\left[Z\right]}{dt}=\;\alpha_{X}\;.\;\left[X\right]+\;\alpha_{Y}\;.\left[Y\right]-\;\delta_{Z}\;.\;\left[Z\right]\\ \frac{d\left[C\right]}{dt}=\;\alpha_{B}\;.\left[B\right]-\;\gamma_{AC}\;.\;\left[A\right]\;.\;\left[C\right]-\;\delta_{C}\;.\;\left[C\right] \end{array}$$

Two IFFL-modified circuits stood out among all the tested designs in this study. The first one features one IFFL circuit between genes *C* and *A*, to form an activation pathway from gene *C* to gene *A,* as shown in figure 1*e*, and is named as 1IFFL-based circuit. This design seems to share the same intention as in the CFFL-based circuit to enhance the interaction between genes *A* and *C*. The second IFFL-based circuit features two IFFL circuits, named as 2IFFL-based circuit, with one in the gene *A* to gene *B* activation pathway, and the other in the gene *B* to gene *C* activation pathway. Different from the 1IFFL-based circuit, the 2IFFL-based circuit seems to emphasize accelerating the response of genes *B* and *C* in affecting the overall dynamics of the circuit. The detailed ODE models for these two designs are given in equations (2.3) and (2.4), respectively. Note, in the 2IFFL-based circuit, ‘*X*1’ and ‘*X*2’ refer to the *X* species in the IFFL between *A* and *B*, and the IFFL between *B* and *C,* respectively. The same applies to genes *Y* and *Z*.


(2.3) $$\begin{array}{l} \frac{d\left[A\right]}{dt}=\;\mu +\alpha_{Z}.\;\left[Z\right]-\;\gamma_{AC}\;.\;\left[A\right]\;.\;\left[C\right]\;-\;\delta_{A}\;.\;\left[A\right]\\ \frac{d\left[B\right]}{dt}=\;\alpha_{A}\;.\;\left[A\right]-\;\delta_{B}\;.\;\left[B\right]\;\\ \frac{d\left[X\right]}{dt}=\;\alpha_{C}\;.\;\left[C\right]-\;\;\delta_{X}\;.\;\left[X\right]\;\\ \frac{d\left[Y\right]}{dt}=\;\alpha_{X}\;.\;\left[X\right]-\;\beta_{Y}\;.\left[Z\right]\left[Y\right]-\;\;\delta_{Y}\;.\;\left[Y\right]\\ \frac{d\left[Z\right]}{dt}=\;\alpha_{X}\;.\;\left[X\right]-\;\beta_{Y}\;.\left[Z\right]\left[Y\right]-\;\delta_{Z}\;.\;\left[Z\right]\\ \frac{d\left[C\right]}{dt}=\;\alpha_{B}\;.\left[B\right]-\;\gamma_{AC}\;.\;\left[A\right]\;.\;\left[C\right]-\;\delta_{C}\;.\;\left[C\right] \end{array}$$

(2.4) $$\begin{array}{l} \frac{d\left[A\right]}{dt}=\;\mu +\alpha_{Z}.\;\left[Z\right]-\;\gamma_{AC}\;.\;\left[A\right]\;.\;\left[C\right]\;-\;\delta_{A}\;.\;\left[A\right]\\ \frac{d\left[B\right]}{dt}=\;\alpha_{Z}\;.\;\left[Z1\right]\;-\;\delta_{B}\;.\;\left[B\right]\\ \frac{d\left[X1\right]}{dt}=\;\alpha_{A}\;.\;\left[A\right]-\;\;\delta_{X}\;.\;\left[X1\right]\;\\ \frac{d\left[Y1\right]}{dt}=\;\alpha_{X}\;.\;\left[X1\right]-\;\beta_{Y}\;.\left[Y1\right]\left[Z1\right]-\;\;\delta_{Y}\;.\;\left[Y1\right]\\ \frac{d\left[Z1\right]}{dt}=\;\alpha_{X}\;.\;\left[X1\right]-\;\beta_{Y}\;.\left[Y1\right]\left[Z1\right]-\;\delta_{Z}\;.\;\left[Z1\right]\\ \frac{d\left[C\right]}{dt}=\;\alpha_{Z}\;.\left[Z2\right]-\;\gamma_{AC}\;.\;\left[A\right]\;.\;\left[C\right]-\;\delta_{C}\;.\;\left[C\right]\\ \frac{d\left[X2\right]}{dt}=\;\alpha_{B}\;.\;\left[B\right]-\;\;\delta_{X}\;.\;\left[X2\right]\;\\ \frac{d\left[Y2\right]}{dt}=\;\alpha_{X}\;.\;\left[X2\right]-\;\beta_{Y}\;.\left[Y2\right]\left[Z2\right]-\;\;\delta_{Y}\;.\;\left[Y2\right]\\ \frac{d\left[Z2\right]}{dt}=\;\alpha_{X}\;.\;\left[X2\right]-\;\beta_{Y}\;.\left[Y2\right]\left[Z2\right]-\;\delta_{Z}\;.\;\left[Z2\right] \end{array}$$

For experimental realization, the FFL motifs can be constructed with RNA regulators such as small transcriptional activating RNA (STAR), and toehold switches [20,52,54], and the sequestration between species *A* and *C* can be designed with antisense RNAs [19,55]. The engineered FFL motifs can then be integrated into the system to be controlled by incorporating at least one identified gene, expressing either a fluorescent protein (GFP) or proteins quantifiable through SDS-PAGE [56,57].

### 2.2. Controller performance evaluation

The dynamics of a gene circuit can be affected by the kinetic parameters associated with the embedded reactions, as they could fluctuate in a real system due to changes in cellular resources and environmental disturbances, such as the transcription and the degradation rates (i.e. α and δ in our ODE models). For example, the availability of polymerase would affect the transcription rate, and ambient temperature could also affect the transcription rate of heat-sensitive promoters. Therefore, here, we quantify the circuit response subject to parameter perturbations. Furthermore, we consider two sets of nominal parameter values: (i) a generic set of parameters, which does not necessarily bear biological relevance but have been widely adopted in mathematical analysis of gene circuits dynamics [6,21,58]. With this effort, we aim to complement existing analysis from a different perspective on antithetic controllers, and more importantly to gain an assessment of the circuit properties with less constricted requirements on the kinetic parameters, given the possibility that novel regulatory parts would emerge to make it feasible to realize currently seemingly unfeasible parameter values; and (ii) a set of parameters obtained from fitting to experiments in previous studies [47,59]. With this effort, we strive to evaluate the characteristics of these circuits for potential practical application, given existing synthetic gene regulatory tools.

To analyse the performance of the circuits, we introduced a step change in the inducer concentration at time zero and adopted the same four standard step-change response analysis metrics from our previous work [38]: *rise time*, which quantifies the responsiveness of the system to a change in the environment; *overshoot*, which measures the difference between the maximum of a system’s response to changes in the environment from its final steady-state value (if a steady state is to be achieved) during the transient period; *settling time*, which characterizes the speed at which a system settles after the disturbance; and *relative steady-state error*, which quantifies the capability of tracking the reference at steady state (if a steady state is to be achieved). We anticipate the four metrics to capture both transient and steady-state behaviours of the system, with rise time and settling time to provide a measure of the dynamic response of the system to environmental changes, and the overshoot together with steady-state error to shed light on the adaptation and robustness of the system. To evaluate their capability of disturbance adaptation, we also perturbed the output concentration with a step change after the system reached steady state, and quantified the steady-state error before and after the perturbance, as well as the settling time as defined above. For illustration purpose, results for perturbation of a 10% increase in the output concentration are presented in this article, while results for higher perturbances (50% and 100%) included in the electronic supplementary material, which aim to evaluate the robustness of each circuit.

### 2.3. Circuit performance with generic kinetic parameters

We first examined the dynamics of the controllers by varying the transcription and degradation rates of the non-FFL species within 0.1–10 times the nominal parameter values given in electronic supplementary material, table S1a, range consistent with previous works [2,5,6], whereas inducer μ=100 M h^−1^, gene *B* activation rate $\alpha_{B}=10$ h^−1^ and sequestration rate $\gamma_{AC}=1\times 10^{3}$ M^−1^ h^−1^, were kept constant. This design of analysis is based on previous findings that the activation and degradation rates dominantly affect the circuit stability [6]. The transcription, repression and degradation rates associated with the FFL circuits were tuned to ensure the system would reach a steady state. The detailed information on the kinetic parameters is provided in electronic supplementary material, table S1.

**Figure 2.**
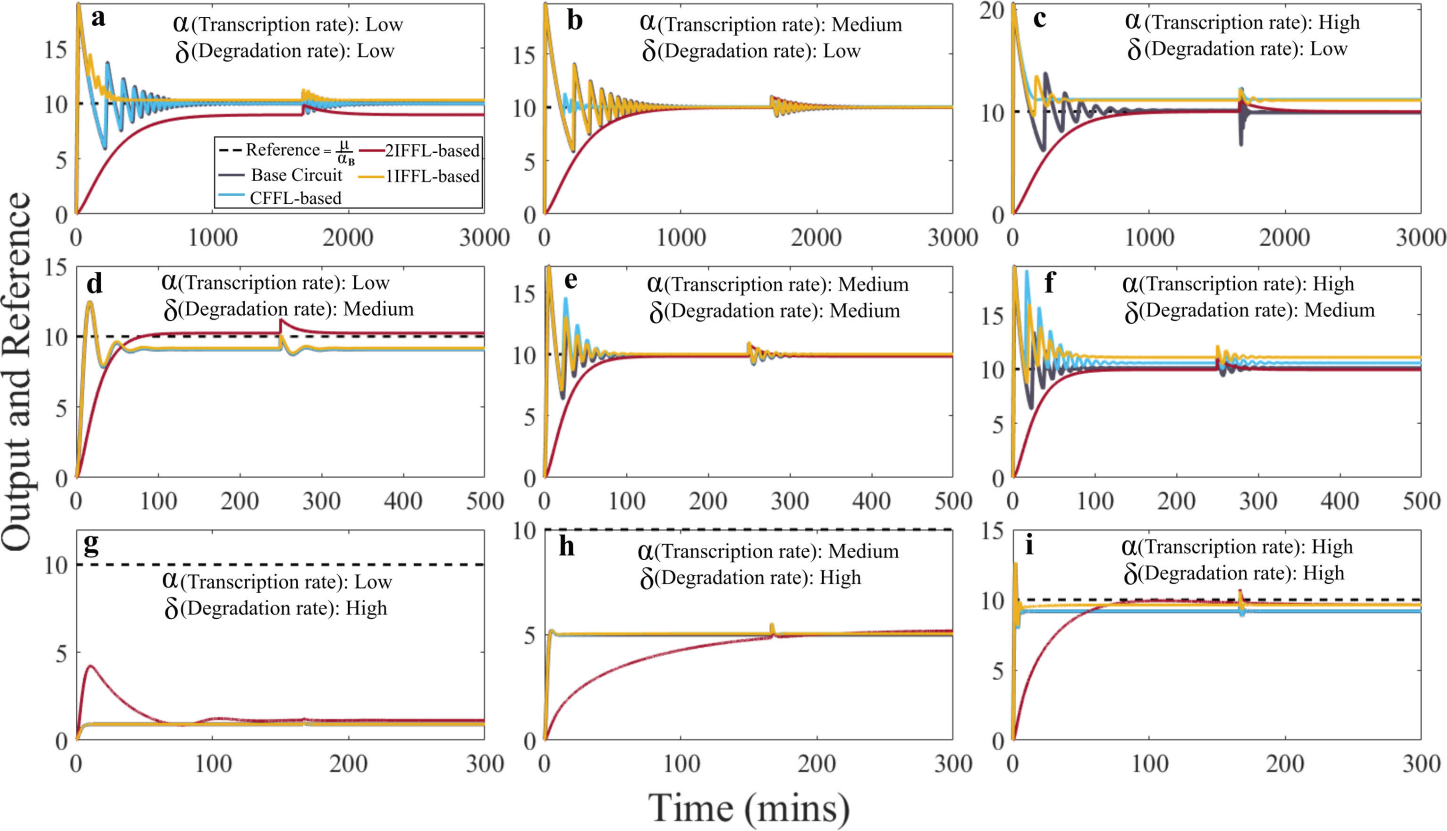
Controller performance to varying transcription and degradation rates, with different scenarios: *Low rate*: $\alpha_{A}=\;\alpha_{C}=1$ h^−1^, $\delta_{A}=\delta_{B}=\delta_{C}=0.1$ h^−1^; *Medium rate*: $\alpha_{A}=\;\alpha_{C}=10$ h^−1^, $\delta_{A}=\delta_{B}=\delta_{C}=1$ h^−1^ *High rate*: $\alpha_{A}=\;\alpha_{C}=100$ h^−1^, $\delta_{A}=\delta_{B}=\delta_{C}=10$ h^−1^.

As shown in figure 2, in response to the step change at time zero, the 1IFFL-based circuit demonstrated the fastest settling to a steady state after around 300 min, for low transcription and degradation rates (figure 2*a*), while the 2IFFL-based circuit provided the slowest response despite the only one that displayed no oscillatory transient in the output concentration (figure 2*d*). The addition of the CFFL motif introduced more oscillatory transient as compared with the base circuit, except for low degradation rates cases (figure 2*a–c*), whereas the IFFL motif tends to reduce the oscillatory transient behaviour in the circuit, with the 2IFFL motifs displaying almost no oscillatory transient behaviour, albeit at the cost of a slower response to the inducer concentration change. An increase in transcription rate α (from the left to the right plots) leads to an increased responsiveness and steady-state gene *B* expression in the base circuit, the CFFL-based circuit and the 1IFFL-based circuit, with an amplified overshoot and oscillatory transient dynamics (figure 2*d*–*f*). However, increasing the transcription rate resulted in similar responsiveness and gene *B* steady-state expression in the 2IFFL-based circuit, showing robust dynamics to the other three circuits. These opposing behavioural outcomes arise as a result of the positioning and configuration of the FFLs. Even though placing an IFFL before gene *B* attenuates the impact of increase in gene *A* on gene *B* expression, an additional IFFL placed before gene *C* offers a compensation of this impact by attenuating gene *C* expression, which increases the sequestration between gene *C* and *A*. Placing an IFFL between genes *B* and *C* attenuates impact on gene *C* due to gene *B* expression increase, as a result, the sequestration between genes *C* and *A* would dominate the dynamics that as the transcription rate increases, gene *B* expression decreases.

While all four circuits demonstrated robustness to variations in transcription rate in terms of adaptation, significant variations in degradation rate (figure 2*g*,*h*) resulted in obvious deviation from the reference. Interestingly, while increasing the degradation rate leads to decreased expression of gene *B*, a combination of high transcription and degradation rates leads to a fast and precise adaptation in the base circuit, CFFL-based and IFFL-based, while 2IFFL-based presents a precise but slower performance (figure 2*i*). This outcome could be attributed to the competition between the IFFL impact on gene *B* production and the sequestration reaction between genes *A* and *C* in the 2IFFL-based circuit, as gene *A* concentration would positively correlate with gene *B* production, but the IFFL between genes *A* and *B* tends to attenuate such activation effect.

When subjected to the perturbation introduced, all circuits demonstrated perfect adaptation, in terms of settling to the original steady state before the disturbance. Except for the 2IFFL-based circuit, all the other three circuits exhibited oscillatory behaviour before regaining the steady state, as shown in figure 2. These observations indicate that adding the IFFL circuits to the base antithetic controller could attenuate the oscillatory behaviour and mitigate the impact on the steady-state concentration subject to variation in certain kinetic parameters, such as the degradation rate, while the addition of the CFFL circuit would potentially introduce more pronounced oscillations.

### 2.4. Circuit performance with experimentally fitted kinetic parameters

While the circuit analysis with parameters that are less confined by biological relevance could provide a more general understanding of its dynamics, such kinetics might not be achievable in experiments, given the current development of gene regulatory tools. As the goal of designing synthetic gene circuits is to deliver desired functions in real biological systems, analysis with biologically relevant parameters would become crucial. Therefore, for the subsequent sections, we focus our analysis on kinetic parameters inferred from previously experimentally fitting [47,59]. The detailed information on the kinetic parameters is provided in electronic supplementary material, table S2.

With parameters from electronic supplementary material, table S2, the CFFL-based circuit demonstrated an unstable behaviour in gene *B* expression, where the output gene expression diverges as time increases (electronic supplementary material, figure S4). This observation differs significantly from the analysis with the non-biologically feasible parameters, but makes sense given that the non-biologically feasible parameters offer more freedom in parameter design that the specific sets of parameters tested did not capture the unstable dynamics observed here. As the addition of the CFFL motif between genes *A* and *C* constructs a competition with the sequestration, depending on the specific kinetics of the sequestration and the CFFL activation pathway, unstable behaviour in gene *B* expression could eventually emerge. Since the CFFL-based circuit did not yield adaptation, its analysis is not included in the following main text but provided in the electronic supplementary material.

**Figure 3.**
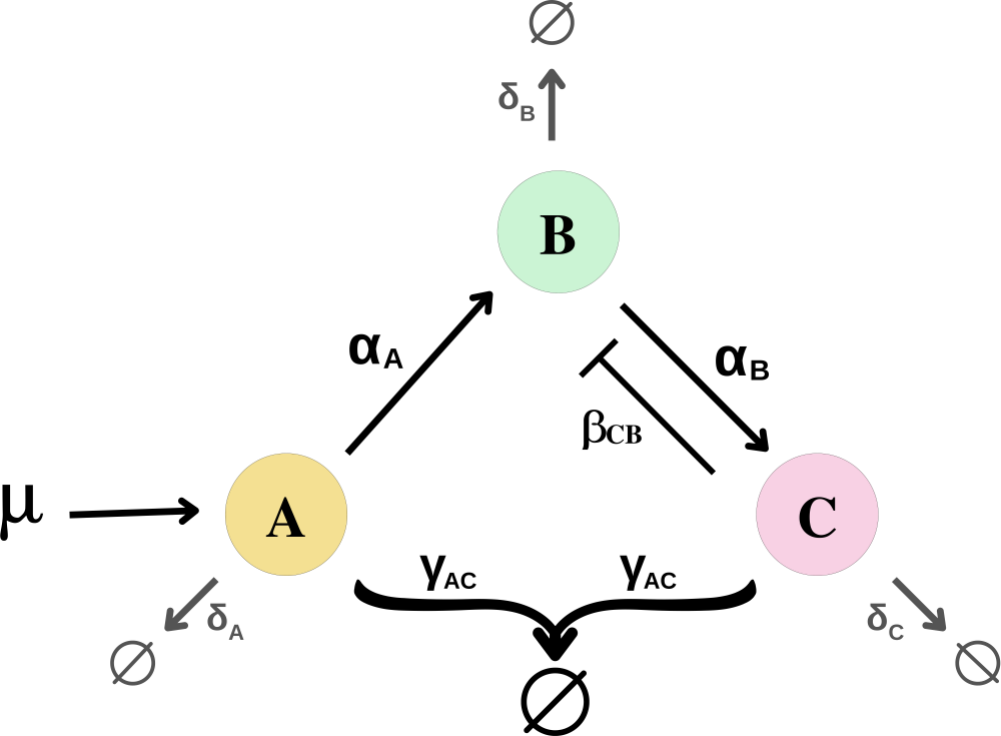
Schematic FB-based circuit, i.e. the antithetic controller with feedback from gene *C* to gene *B*.


As we aim for biological controller designs that deliver excellent transient dynamics as well as reference tracking, but not necessarily (quasi-)integral controllers, we also note that a previously reported antithetic controller with negative feedback from gene *C* to gene *B* (figure 3) could improve the dynamics in mammalian cells [9]. For completeness, we include this design (named as FB-based circuit here) in our analysis along with the remaining two IFFL-based circuits. Instead of the Hill-type function modelling considered in Gupta & Khammash [9], here, we develop a simple ODE model given in equation (2.5), to represent the FB-based circuit for a consistent comparison with the other circuits.

(2.5) $$\begin{array}{l} \frac{d\left[A\right]}{dt}=\;\mu -\;\gamma_{AC}\;.\;\left[A\right]\;.\;\left[C\right]\;-\;\delta_{A}\;.\;\left[A\right]\\ \frac{d\left[B\right]}{dt}=\;\alpha_{A}\;.\;\left[A\right]-\beta_{CB}\;.\left[B\right].\;\left[C\right]-\;\delta_{B}\;.\;\left[B\right]\\ \frac{d\left[C\right]}{dt}=\;\alpha_{B}\;.\;\left[B\right]\;-\beta_{CB}\;.\left[C\right].\;\left[B\right]-\;\gamma_{AC}\;.\;\left[A\right]\;.\;\left[C\right]-\;\delta_{C}\;.\;\left[C\right] \end{array}$$

### 2.5. Analysis with varying transcription and degradation rates

**Figure 4.**
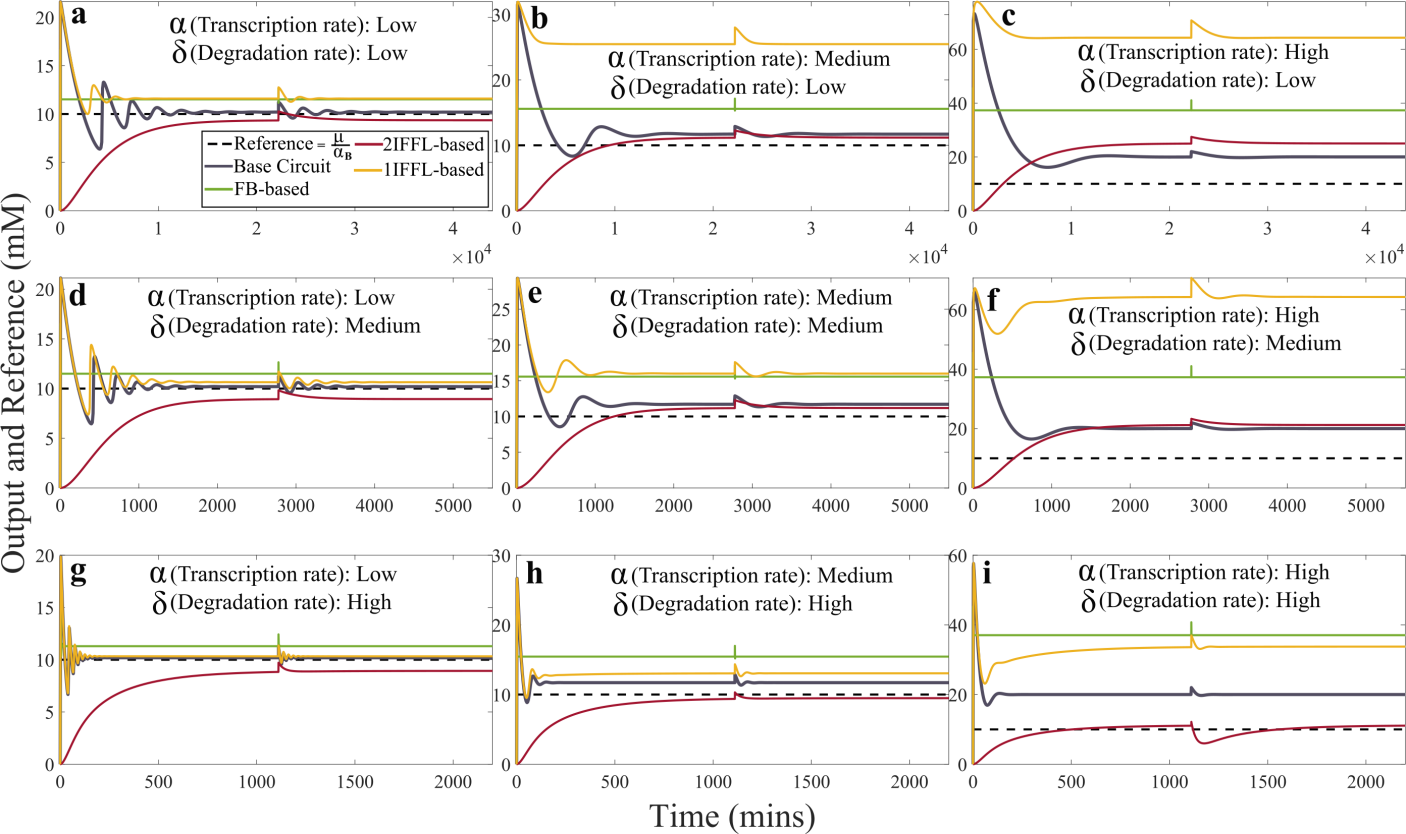
Controller performance to varying transcription and degradation rates, with previous experimentally fitted parameters and: *Low rate*: $\alpha_{A}=\;\alpha_{C}=10^{-2}$ s^−1^, $\delta_{A}=\delta_{B}=\delta_{C}=10^{-5}$ s^−1^; *Medium rate*: $\;\alpha_{A}=\alpha_{C}=10^{-1}\;s^{-1},\;\delta_{A}=\delta_{B}=\delta_{C}=10^{-4}\;s^{-1}$; *High rate*: $\alpha_{A}=\alpha_{C}=1\;s^{-1}$, $\delta_{A}=\delta_{B}=\delta_{C}=10^{-3}\;s^{-1}$. Inset figure in (*a*) shows the fast transient dynamics of the FB-based circuit.

To be consistent with the analysis with the generic parameters, we first analysed the circuit dynamics by varying only the transcription and degradation rates within 0.1–10 times the nominal parameter values given in electronic supplementary material, table S2. The inducer $\mu =10^{-7}$ M s^−1^, sequestration rate $\gamma_{AC}=5\times 10^{5}$ M^−1^ s^−1^, gene *B* transcription rate $\alpha_{B}=\;10^{-1}$ M^−1^ s^−1^ and the repression from gene *C* to gene *B*, $\beta_{CB}=10^{5}$ M^−1^ s^−1^, were all kept constant. The results are summarized in figure 4.

Both the base circuit (black plots) and the 2IFFL-based circuit (red plots) showed remarkable adaptation under the tested scenarios, especially when the transcription rate increases, and for the 2IFFL-based circuit, the impact of a high transcription rate can be mitigated by a high degradation rate to ensure perfect adaptation (figure 4*i*). Moreover, in all the scenarios, the base circuit demonstrated a rapid and oscillatory response to the stimuli before settling to the steady state, while the 2IFFL-based circuit showed no oscillation, but with a slow ramping increase in the gene *B* expression until reaching the steady state. Surprisingly, the FB-based circuit (green plots) failed to achieve perfect adaptation in all nine scenarios but demonstrated the fastest response to stimuli change without oscillation. This might be because the feedback interaction is modelled as a consumption between genes *B* and *C* here, whereas a Hill-type repression function could potentially mitigate the aggressive dynamics [9]. Also contrary to our expectation, the 1IFFL-based circuit (yellow plots) did not yield adaptation in most scenarios, except for scenarios with low transcription rates combined with medium and high degradation rates (figure 4*d*,*g*), and also demonstrated significant transient oscillatory behaviour. To complement the transient dynamics analysis, we also performed equilibrium analysis for each of the circuits, subjecting to different transcription and degradation rates in electronic supplementary material, figure S9. We noticed that the base circuit displayed minimal sensitivity to changes in the degradation rate, whereas both the 1IFFL-based and 2IFFL-based circuits demonstrated significant dependence on both the transcription and degradation rates. The detailed results and discussions are provided in the electronic supplementary material.

### 2.6. Global sensitivity analysis for statistical analysis

To gain a more statistically significant understanding of the four circuits, we then performed a global sensitivity analysis with 1000 simulations, where all the model kinetic parameters were randomly perturbed within 0.1× and 10× of their corresponding nominal values given in electronic supplementary material, table S2. For this sensitivity analysis, no perturbation was introduced after the system reached the steady state.

Of the 1000 simulations, 996, 947, 648 and 1000 simulations reached steady state after the initial 2000 min, for the base circuit, the 1IFFL-based circuit, the 2IFFL-based circuit and the FB-based circuit, respectively. This observation indicates that both the base circuit and the FB-based circuit are robust to variations in the kinetic parameters, whereas incorporating the IFFL motif could undermine such robustness, in terms of converging to a steady state. This is probably due to the increased complexity in the IFFL-modified circuits. Moving forward, we focused our analysis on these simulations that have reached a steady state for each circuit.

**Figure 5.**
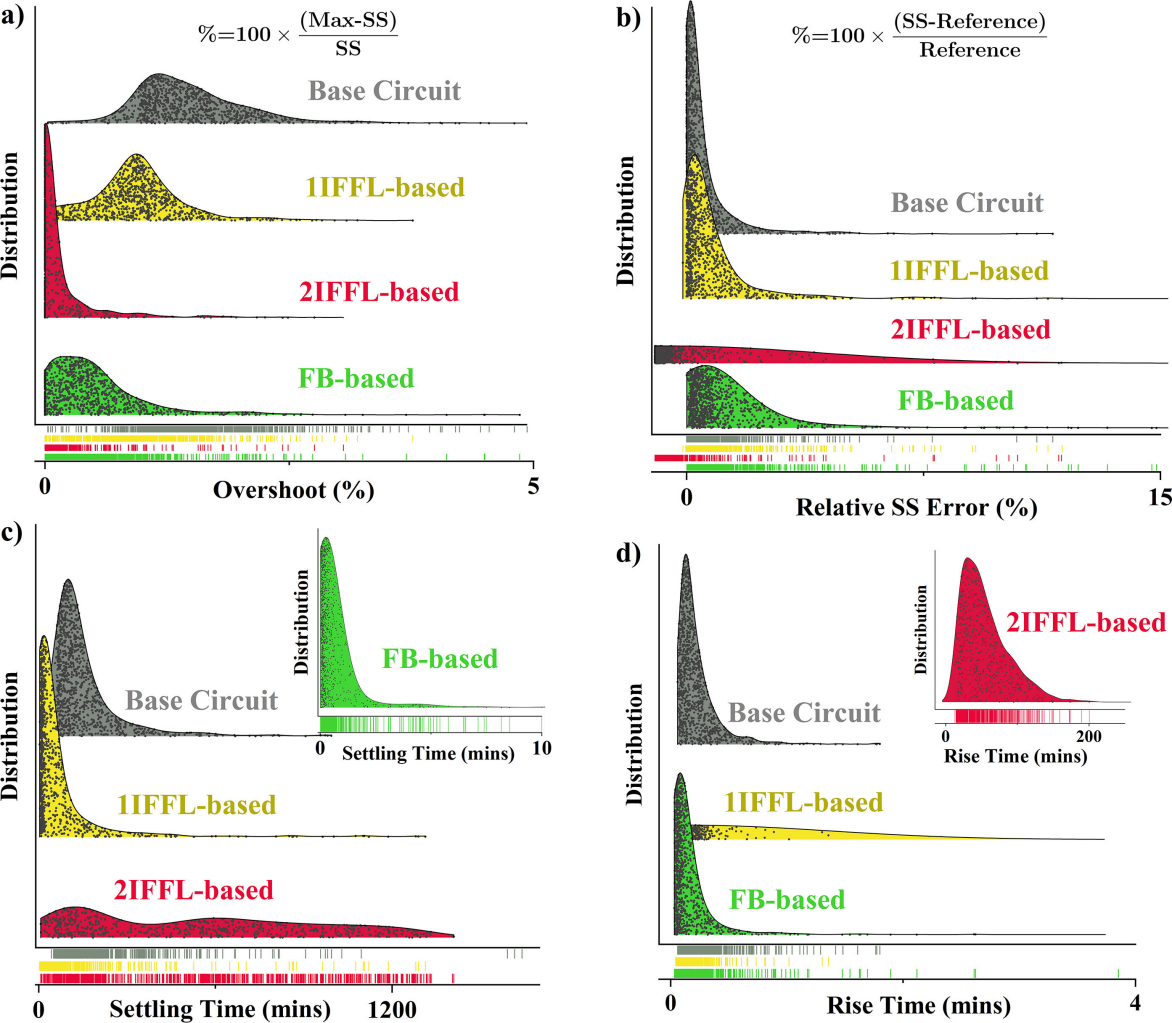
Global sensitivity analysis with simulations reached steady state without a second perturbation, in terms of: (*a*) overshoot; (*b*) relative steady-state error; (*c*) settling time; and (*d*) rise time. The dots beneath each plot show the location of all the data on the *x*-axis.

The distributions of overshoot, relative steady-state error, settling time and rise time after the step change at time 0 in figure 5 demonstrate that: (i) the two IFFL-based circuits and the FB-based circuit all endowed a smaller overshoot as compared with the base circuit, indicated by the left-skewed distribution of the overshoot in figure 5*a*, with the 2IFFL-based circuit showing the lowest overshoot. (ii) The FB-based circuit demonstrated the best performance in terms of relative steady-state error, and both the 1IFFL-based and the FB-based circuits also showed similarly small relative steady-state error. While the 2IFFL-based circuit also yielded small relative steady-state error, it tends to yield a lower than reference steady-state value (thus a negative steady-state error) rather than a higher than steady-state value as observed in the other three circuits. (iii) The FB-based circuit showed the best performance in terms of rapid settling time and fast response to stimuli (rise time) as depicted in figure 5*c*,*d*. While the base circuit showed a comparable responsiveness to stimuli (rise time) in figure 5*d*, the settling time could span from minutes to hundreds of minutes, depending on the specific values of the kinetic parameters as shown in figure 5*c*. On the other hand, while the 1IFFL-based circuit improved the settling time as compared with the base circuit, the addition of the IFFL motif resulted in a wide distribution of the settling time as in figure 5*d*. This is more prominent with the 2IFFL-based circuit, where both the rise time and the settling time span from minutes to hundreds of minutes, suggesting a slower dynamic compared with the base and the FB-based circuits.

**Figure 6.**
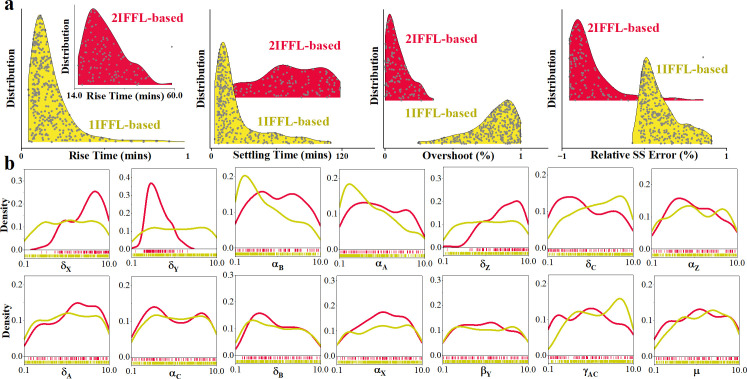
Desired kinetic parameter ranges for antithetic controller with IFFL-based circuits to deliver a rapid response and settling to changes in inducer stimuli, with mitigated overshoot and adaptation. (*a*) Performance metric distribution comparison of 1IFFL-based and 2IFFL-based circuits. (*b*) Kinetic parameter densitiy for 1IFFL-based (yellow) and 2IFFL-based (red) circuits to achieve the desired performance.

Further analysis on the kinetic parameter requirements for the two IFFL-based circuits to achieve a rapid response (rise time ≤60 mins), moderate overshoot (|overshoot| ≤1%), fast adaptation (settling time ≤120 mins) and reasonable relative steady-state error (|error| ≤1%), reveals a higher flexibility in designing the parameters for the 2IFFL-based circuit, as compared with the 1IFFL-based circuit. Specifically, as indicated in figure 6, the 2IFFL-based circuit (red plot in figure 6*b*) would require a high degradation rate for genes *B* ($\delta_{X}$) and *Z* ($\delta_{Z}$), but a low degradation of gene *Y* ($\delta_{Y})$, gene *C* ($\delta_{C})$ and *B* ${(\delta }_{B})$, showing no obvious preference in the remaining nine parameters. In contrast, the 1IFFL-based circuit (yellow plot in figure 6*b*) would require a high degradation rate of gene *C* ($\delta_{C}$), but a low transcription rate of genes *A* $\left(\alpha_{A}\right)$ and *B* $\left(\alpha_{B}\right)$, as well as a relatively higher sequestration rate between genes *A* and *C* ($\gamma_{AC}$), with no obvious preference in the remaining 11 parameters. It is noteworthy that the parameters employing the most significant influence on the circuit are limited to transcription and degradation rates. This observation aligns with the stability analysis elucidated by Olsman *et al*. [6], which asserts that the stability of the antithetic integral type of circuits is dominated by the values of transcription and degradation rates.

**Figure 7.**
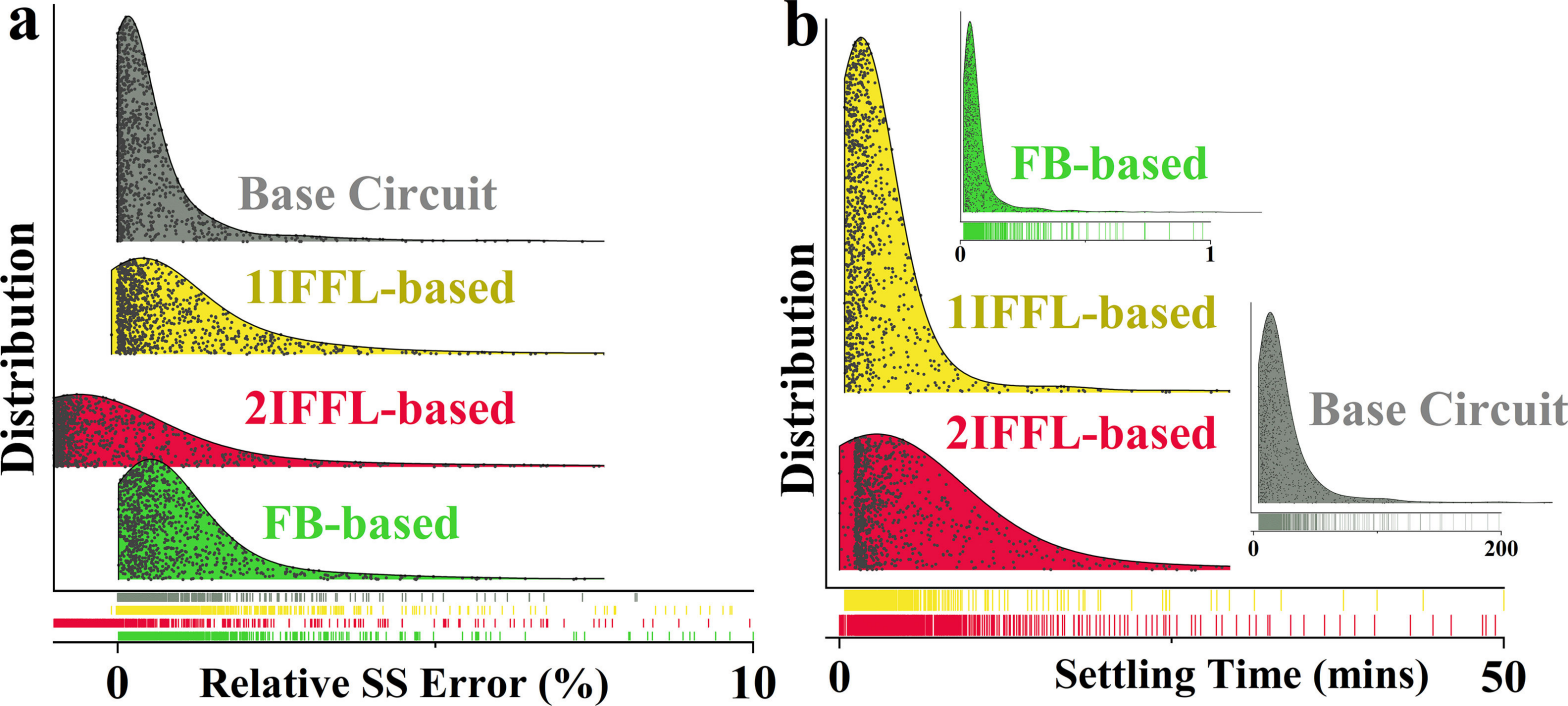
Global sensitivity analysis for simulations after disturbance in terms of: (*a*) relative steady-state error; and (*b*) settling time. 10% Perturbation.

We also performed a sensitivity analysis for simulations with secondary perturbation (10%) after reaching the steady state in figure 7. The result demonstrates that, while all four circuits yielded comparable adaptation, the relative percentage steady-state error in figure 7*a* shows that the FB-based circuit exhibited the fastest settling time, whereas the base circuit showed the slowest settling dynamics. This observation indicates that the direct feedback pathway and the addition of IFFL circuits could accelerate the transient dynamics in the disturbance adaptation of the base circuit.

## 3. Conclusions

In this work, we investigated the dynamics of different antithetic controllers modified with the FFL biological motifs and evaluated the benefits and limitations of such modifications through simulation. Our results demonstrate that while the base circuit excels in adaptation and rapid stimuli response, its oscillatory dynamics with significant overshoot could have resulted from the disturbances in the inducer. The introduction of a negative feedback to repress the expression of the target gene could contribute to mitigated overshoot with significantly enhanced response to stimuli, demonstrated by the rapid rise time and settling time in the FB-based circuit. However, such an aggressive interaction could result in compromised adaptation. In contrast, adding the IFFL motif to the base circuit could in general effectively attenuate the oscillatory dynamics and mitigate unwanted overshoot due to changes in the inducer concentration. However, analysis with biologically feasible parameters suggests that circuits modified with the IFFL motif could potentially slow down the response to stimuli changes, and the mitigation and elimination of the oscillation could also result in a slow adaptation to steady state. These phenomena would probably be due to the increased complexity in the design as a result of adding the IFFL motifs to the base circuit. To achieve a circuit with a fast response and settling time, as well as a moderate overshoot and mitigated oscillation, a delicate design of the kinetic parameters would be necessary.

Given the prevalence of the IFFL motif in natural biological systems, as well as the development of novel synthetic designs of the IFFL circuits [60–63], we expect a wider application of the IFFL circuits in more complicated biological regulators for enhanced functionalities. The performance of a synthetic gene circuit in real system implementation is always undermined by cellular resource competition with native biological activities, thus a design with light cellular burden is normally preferred. However, this typically comes at the price of limited functionality. Recent advancement in the division of labour [48,64,65], where a complicated circuit is split into parts and implemented in different groups or species of cells to carry out independent activities and communicate through quorum sensing to deliver the anticipated overall function, was found capable of reducing cellular burden and mitigating the impact of resource competition on the performance. We anticipate research efforts in this direction, as well as on leveraging resource competition as a negative control to cooperatively regulate the circuit dynamics, to facilitate the implementation of a complex circuit on real biological systems.

The work presented here offers a computational analysis of different alternative antithetic controllers and serves as a preliminary step towards the design of novel synthetic circuits with advanced functions using existing biological motifs. While analysis with less emphasis on the biological relevance could offer a general understanding of the circuit properties, especially with analytical studies on the architectural properties [2,8,21,66] as the circuit dynamics is significantly affected by the specific kinetic parameter values, discrepancies could arise when biological relevance is considered. This suggests that for practical applications, analysis centred around biologically feasible conditions is vital in guiding the design and construction of novel circuits. As the circuit dynamics is context-dependent, experimental characterization of basic genetic regulatory parts under different conditions could offer a more reliable range of biological feasible parameter values, to facilitate the simulation-based analysis, and in return, to accelerate the exploration of new circuits with enhance performances. In future research, we plan to validate the proposed IFFL-based antithetic controller in both *E. coli* and cell-free systems, to gauge the performance in experiments, as well as the reliability of model-based analysis. We envision such efforts to further contribute to our understanding on building novel circuits with natural biological regulatory motifs for advanced functionalities, and to the experiment–simulation integrated exploration for new synthetic circuits.

## 4. Methods

### 4.1. Mathematical models and simulation

The ODE-based models were constructed for each antithetic controller design and solved utilizing the MATLAB ode23s solver. These models accounted for species interactions involving activation, degradation, sequestration and repression, with kinetic parameters derived from established literature in the field. The sensitivity analysis was executed by systematically altering all kinetic parameters within the model. In each simulation, the designated kinetic parameters for each circuit were multiplied by a ‘scaling factor’, spanning from 10^-1^ to 10. This approach facilitated a thorough exploration of the parameter space, providing insights into the models’ sensitivity to variations in kinetic parameters. 1000 simulations were performed for each genetic circuit in the sensitivity analysis, and the violin plots presented in figure 5 were generated using Origin Pro.

## Data Availability

All the MATLAB scripts generated in this study are deposited on Zenodo and can be found here [67]. Supplementary material is available online [68].
